# Transcriptomic Analysis Identifies Complement Component 3 as a Potential Predictive Biomarker for Chemotherapy Resistance in Colorectal Cancer

**DOI:** 10.3389/fmolb.2021.763652

**Published:** 2021-10-15

**Authors:** Xiao-Shun He, Sheng-Yi Zou, Jia-Lu Yao, Wangjianfei Yu, Zhi-Yong Deng, Jing-Ru Wang, Wen-Juan Gan, Shan Wan, Xiao-Qin Yang, Hua Wu

**Affiliations:** ^1^ Department of Pathology, Medical College of Soochow University and The First Affiliated Hospital of Soochow University, Soochow University, Suzhou, China; ^2^ Department of Endocrinology and Metabolism, The First Affiliated Hospital of Soochow University, Soochow University, Suzhou, China; ^3^ Department of Cardiology, the First Affiliated Hospital of Soochow University, Soochow University, Suzhou, China; ^4^ Department of Bioinformatics, Medical College of Soochow University, Soochow University, Suzhou, China; ^5^ Department of Pathology, The First People’s Hospital of Kunshan, Kunshan, China; ^6^ Department of Pathology, Dushu Lake Hospital Affiliated of Soochow University, Soochow University, Suzhou, China

**Keywords:** colorectal cancer, C3, oxaliplatin resistance, prognosis, transcriptomic analysis

## Abstract

**Objective:** 5-fluorouracil- and oxaliplatin-based FOLFOX regimens are mainstay chemotherapeutics for colorectal cancer (CRC) but drug resistance represents a major therapeutic challenge. To improve patient survival, there is a need to identify resistance genes to better understand the mechanisms underlying chemotherapy resistance.

**Methods:** Transcriptomic datasets were retrieved from the Cancer Genome Atlas (TCGA) and Gene Expression Omnibus (GEO) databases and combined with our own microarray data. Weighted gene co-expression network analysis (WGCNA) was used to dissect the functional networks and hub genes associated with FOLFOX resistance and cancer recurrence. We then conducted analysis of prognosis, profiling of tumor infiltrating immune cells, and pathway overrepresentation analysis to comprehensively elucidate the biological impact of the identified hub gene in CRC.

**Results:** WGCNA analysis identified the complement component 3 (C3) gene as the only hub gene associated with both FOLFOX chemotherapy resistance and CRC recurrence after FOLFOX chemotherapy. Subsequent survival analysis confirmed that high C3 expression confers poor progression-free survival, disease-free survival, and recurrence-free survival. Further correlational analysis revealed significant negative association of C3 expression with sensitivity to oxaliplatin, but not 5-fluorouracil. Moreover, *in silico* analysis of tumor immune cell infiltration suggested the change of C3 expression could affect tumor microenvironment. Finally, gene set enrichment analysis (GSEA) revealed a hyperactivation of pathways contributing to invasion, metastasis, lymph node spread, and oxaliplatin resistance in CRC samples with C3 overexpression.

**Conclusion:** Our results suggest that high C3 expression is a debilitating factor for FOLFOX chemotherapy, especially for oxaliplatin sensitivity, and C3 may represent a novel biomarker for treatment decision of CRC.

## Introduction

Colorectal cancer (CRC) is a critical global medical issue. While CRC cases and deaths have decreased over the last decades due to improved screening efforts and novel therapies, it has been estimated that CRC still ranked third in overall morbidity and mortality in the United States in 2021. The prognosis of CRC patient is strongly dependent on the clinical stage at diagnosis: the 5-years survival rate for stage I is 90%, compared with only 10% for stage IV (Virk et al., 2019). Surgery is the main treatment option during the early stages of colorectal cancer, but unfortunately, in many cases the disease is only detected in advanced stages or when distant metastasis has already occurred. Therefore, the discovery and establishment of new strategies for early diagnosis of colorectal cancer will be of great clinical significance.

CRC is a heterogeneous disease with a complex etiology involving an interplay of defective DNA damage repair, somatic mutations, chromosomal instability, microsatellite instability, DNA methylation, and epigenetic alterations (Colussi et al., 2013; Turano et al., 2019; Xu et al., 2021), all of which are thought to contribute to the malignant transformation of intestinal epithelial cells. These factors may also influence treatment response and prognosis of CRC patients. Transition from adenoma to adenocarcinoma is the classic model of CRC progression. Moreover, certain genetic alterations have been shown to be involved in the progression of CRC (Vogelstein et al., 2013). For instance, *APC* mutations are thought to occur first, followed by *KRAS* mutation and subsequent *TP53* inactivation, resulting in abnormal activation of the Wnt/β-catenin, PI3K/Akt, NF-κB, JAK/STAT, and TGF-β/BMPs pathways (Prasad et al., 2010; Fearon, 2011; Vogelstein et al., 2013; Groner and von Manstein, 2017). Many genes such as *APC, TP53, PIK3CA, SMAD4, FBXW7, BRAF, KRAS, and HER-2*, amongst others, have been found to be commonly mutated in CRC (Muzny et al., 2012). Interestingly, *FBXW7*, *HER-2* and *TP53* have been found to be more frequently mutated during the early stages than in advanced stages (Testa et al., 2018; Yaeger et al., 2018). Moreover, *TP53* and *CTNNB1* mutations are thought to more frequently occur in younger patients while mutations in *APC*, *KRAS*, *BRAF*, and *FAM123B* are more likely to occur in older patients (Lieu et al., 2019). The liver is the most common organ for CRC metastasis, with at least 25% CRC patients exhibiting liver metastases following tumor progression (Martin et al., 2020). The main genetic mutations associated with CRC metastasis are mutations in *KRAS* and *TP53*, although some studies have found that *SMAD4* and *BRAF* may also play an important role in CRC metastasis. We recently reported that TRAF6 can inhibit epithelial-mesenchymal transformation and CRC metastasis by driving selective β-catenin degradation via autophagy (Wu et al., 2019), while truncated RXRα (tRXRα) promotes colitis-associated colorectal tumorigenesis via activation of NF-κB-IL-6-STAT3 signaling in mouse (Ye et al., 2019). We recently also reported that the long non-coding RNA NONHSAT062994 acts as a tumor suppressor and inhibits CRC development via inactivation of Akt signaling (He et al., 2017).

The identification of circulating biomarkers, such as circulating tumor cells, exosomes, and miRNAs in blood (Turano et al., 2019), for early detection as well as monitoring treatment efficacy, recurrence, and metastasis of CRC has been a focus of research in recent years (Diaz et al., 2012; Tie et al., 2015). A limited number of targeted therapeutics, such as anti-EGFR drugs, have received approval for the treatment of colorectal cancer by the US FDA. However, as KRAS and NRAS act downstream of the EGFR pathway, CRC patients with *KRAS* mutations do not benefit from anti-EGFR therapy (Lieu et al., 2019). The mutation rate of *BRAF* in CRC is 8–13%, with the most common mutation site being V600E. *BRAF* mutation, especially in MSI-L (Microsatellite Instability-Low) and MSS (Microsatellite Stability), can enhance tumor resistance to anti-EGFR drugs, and result in a poor prognosis (Lech et al., 2016). Excitingly, new immunotherapies may elicit remission in more patients. The monoclonal antibodies Pembrolizumab and Nivolumab exert antitumor effects by blocking programmed cell death protein 1 (PD-1). They are suitable for patients with MSI-H (Microsatellite Instability-High) or dMMR (Dificient Mismatch Repair) and can also be used for patients with liver metastases, although patients with MSI-L or pMMR do not benefit from the anti-PD-1 treatment (Turano et al., 2019).

Despite the abovementioned efforts in cancer screening, treatment, and monitoring, the 5-years survival rate of CRC patients is still unsatisfactory. Therefore, the discovery of novel therapeutic strategies and identification of regulatory molecules is urgently needed. In this study, we combined transcriptomic data from public databases with our own work and found that complement component 3 (C3) gene was associated with oxaliplatin resistance via reprogramming the tumor immune microenvironment in CRC. These findings suggest C3 represents a potential predictive biomarker for the prognosis of oxaliplatin-based therapy in CRC patients.

## Materials and Methods

### Data Collection

RNA-Seq transcriptomic data of colon adenocarcinoma patients were retrieved from TCGA-COAD project via the R TCGAbiolinks package (version: 1.12.0) (Colaprico et al., 2015). The corresponding clinical information of the TCGA-COAD samples was obtained from the UCSC Xena database (Goldman et al., 2020). Microarray datasets (GSE39582, GSE81653, GSE87211, GSE28702 and GSE17536) for colorectal cancer samples and matching clinical characteristics were downloaded from the NCBI Gene Expression Omnibus (GEO) database. Microarray datasets for 5-Fluorouracil and oxaliplatin resistant cellular models (GSE76489 and GSE83131) were also obtained from GEO. Gene expression profiles of colorectal cancer cell lines and corresponding half-maximal inhibitory concentration (IC50) of 5-Fluorouracil and oxaliplatin were obtained from the Genomics of Drug Sensitivity in Cancer (GDSC) database (Yang et al., 2013).

### Clinical Specimens

Colorectal cancer (CRC) samples (four with lymph node metastasis and three without lymph node metastasis) were obtained from patients who underwent curative surgery at the First Affiliated Hospital of Soochow University (Suzhou, Jiangsu, P.R. China). All patients included in this study provided written informed consent for participation. The study was approved by the Biomedical Research Ethics Committee of Soochow University (Suzhou 215123, Jiangsu Province, China).

### Microarray Experiment

Total RNA of each sample was extracted using TRIzol reagent. The Agilent Human lncRNA-mRNA Microarray V2.0 4 × 180 K (Agilent Technologies, Inc.) was used to compare transcriptomic profiles between subgroups with and without lymph node metastasis. RNA labeling, microarray hybridization, and data acquisition were performed by Shanghai OE Biotech. Co., Ltd. The raw microarray data reported in this paper has been deposited in the OMIX, China National Center for Bioinformation/Beijing Institute of Genomics, Chinese Academy of Sciences, under the accession number OMIX505 and can be accessed at https://ngdc.cncb.ac.cn/omix.

### High Throughput Data Preprocessing and Assessment of Differentially Expressed Genes

For TCGA-COAD RNA-Seq data, the raw reads count matrix was converted to the counts per million mapped reads (CPM) format. Trimmed mean of M values (TMM) normalization was carried out to correct the variation of gene expression abundance across different samples. Both log2-transformed or raw formats were generated for further bioinformatics analyses. Samples were categorized according to the median expression level of target genes. Differentially expressed genes were filtered using the Benjamini-Hochberg false discovery rate approach (adjusted *p*-value < 0.05, and absolute value of log2 fold-change > 1). All preprocessing and differential gene expression analysis was conducted using an R pipeline based on “limma” (version: 3.48.1) (Ritchie et al., 2015) and “edgeR” (version: 3.34.0) (Robinson et al., 2010) R packages.

For Affymetrix microarray data, data was normalized using the Robust Multi-array Average (RMA) method. For Agilent microarray data, data were normalized using the “quantile” method. Gene level expression estimates were log2-transformed and summarized into a single matrix for subsequent analysis. Raw *p*-values < 0.01 were used as the cutoff to identify differentially expressed genes. All microarray analyses were performed using the “limma” R package (version: 3.48.1).

### Weighted Gene Co-expression Network Analysis

Genes were ranked according to the median absolute division (MAD) values across samples in the GSE81653 and GSE28702 dataset, and the top 5,000 genes were subjected to the subsequent weighted gene co-expression network analysis (WGCNA). Briefly, a minimum power with *R* ^2^ > 0.85 was set to optimize the power (β) for automatic network and further topological overlap matrix construction. After that, minModuleSize = 30 was utilized to initially classify co-expression modules. In one data set with less than 100 samples (GSE28702), the cutHeight was set to 0.2. Conversely, when the sample size exceeded 100 (GSE81653), this parameter was set to 0. A *p*-value less than 0.05 indicated statistical significance for the association between co-expression modules and clinical traits. Module membership (MM) values >0.7 served as the cutoff for key gene screening. The intersection between the results of GSE81653 and GSE28702 was taken for further analysis. All computational analyses were performed using the “WGCNA” R package (version: 1.70-3) (Langfelder and Horvath, 2008).

### Interaction Network Analysis

The STRING database (version 11.0) (Szklarczyk et al., 2019) was used to identify a protein-protein interaction network linked by the target gene connecting the significant gene modules yielded by WGCNA and the up-regulated genes in the colorectal cancer samples with lymph node metastasis (filtered by *p*-value < 0.05 in the “limma” workflow). The “clusterProfiler” R package (version: 4.0.2) (Wu et al., 2021) was used to annotate the biological function of the genes in the network. A raw *p*-value less than 0.05 indicated statistically significant enrichment.

### Gene Set Enrichment Analysis

Gene set enrichment analysis (GSEA) was performed to determine whether certain biological pathways or *a priori* defined gene sets showed statistically significant differences between two subgroups with high and low expression of the target gene. The “clusterProfiler” R package (version: 4.0.2) was used for enrichment analyses (Wu et al., 2021). Gene sets including Chemical and Genetic Perturbations, KEGG pathway, and Gene Ontology Biological Process, were accessed using the Molecular Signatures Database (MSigDB). Gene markers for lymph node metastasis were defined as the top 300 significantly up-regulated genes (*p*-value less than 0.01) ranked by log2-transformed fold change obtained from our own microarray data. A raw *p*-value less than 0.05 indicated statistically significant enrichment.

GSEA was also used to determine whether the gene signatures that changed upon the expression of the target gene showed statistically significant, concordant differences in 5-Fluorouracil or Oxaliplatin resistant and naïve cell lines. Significant differentially expressed genes between the TCGA-COAD subgroups with high and low expression of the target gene were ranked according to the log2-transformed fold change. The top 300 up- and down-regulated genes were defined as the gene sets. GSEA was then applied to examine the enrichment of these gene sets in the GSE76489 (5-Fluorouracil resistance model) and GSE83131 (Oxaliplatin resistance model) datasets, respectively. A raw *p*-value less than 0.05 indicated statistically significant enrichment.

### In Silico Assessment of Chemotherapy Reagent Sensitivity

The Pearson correlation between log2-transformed levels of the target gene expression and ln-transformed IC50 from the GDSC database were calculated. A *p*-value less than 0.05 indicated statistical significance.

### Survival Analysis

We used the GSE39582 dataset for evaluation of recurrence risk. Samples were categorized according to the median expression level of the target gene. Odds ratios (ORs) and corresponding 95% confidence intervals (95% CIs) were calculated to assess the risk of colorectal cancer recurrence. Stratified analyses according to TNM stage, size of primary tumor (T), regional lymph node involvement (N), presence of distant metastatic spread (M), and mutation status of *TP53*, *KRAS*, and *BRAF* were performed to assess whether the effect of the target gene is influenced by the above-mentioned confounding factors.

TCGA-COAD, GSE17536, GSE87211 and GSE39582 datasets were used for prognostic analyses to assess the impact of the target gene on colorectal cancer survival. The optimal cutoff values for each dataset were determined by the algorithms embedded in the “survminer” (version: 0.4.9) R package. The logrank test was performed to compare the survival distributions between the assigned subgroups. A *p*-value less than 0.05 indicated statistical significance.

### Analysis of Tumor Infiltrating Immune Cells

CPM reads of the TCGA-COAD dataset were assessed for immune cell infiltration analysis. Samples were categorized according to the median expression level of the target gene. The cell-type identification by estimating relative subsets of RNA transcripts (CIBERSORT) method (Newman et al., 2015) was used to profile tumor infiltrating immune cells and compare the differences between the two subgroups. A *p*-value less than 0.05 indicated statistical significance.

### Statistical Analysis

All bioinformatics analyses were carried out in the R programming environment (version: 4.1.0).

## Results

### WGCNA Analyses Identifies C3 as the Common Gene Associated With FOLFOX Chemotherapy Resistance and Cancer Recurrence Risk

**FIGURE 1:**
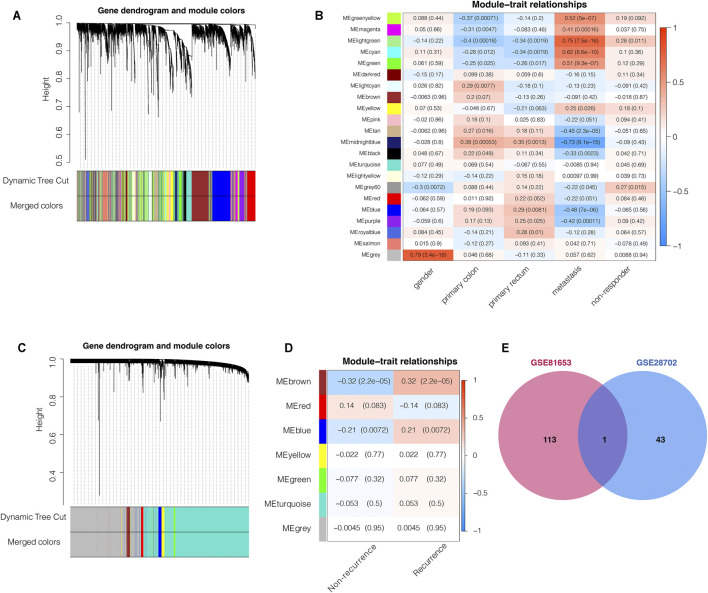
Weighted gene co-expression network analysis (WGCNA) predicts that C3 gene aggravates FOLFOX chemotherapy resistance and recurrence of colorectal cancer. **(A)** Clustering dendrogram of the top 5,000 most variant genes in the GSE28702 dataset, with dissimilarities based on topological overlap labelled with assigned module colors **(B)** Heatmap for the correlation of WGCNA modules with clinical traits (GSE28702). **(C)** Clustering dendrogram of top 5,000 most variant genes in the GSE81653 dataset, with dissimilarities based on topological overlap labelled with assigned module colors **(D)** Heatmap for the correlation of WGCNA modules with clinical traits (GSE81653). **(E)** Venn plot indicated that the C3 gene was the only common element between the chemotherapy respondence-associated module (WGCNA of GSE28702) and the colorectal cancer recurrence-associated module (WGCNA of GSE81653). Genes with module membership values >0.7 were used for this intersection analysis. A *p*-value less than 0.05 indicated statistical significance.

To identify key genes associated with specific clinical traits of colorectal cancer, we employed the WGCNA method to identify gene module-trait correlations. We categorized the top 5,000 genes with the highest MAD in the GSE28702 dataset into 22 co-expression modules (Figure 1A). The MEgrey60 and Melightgreen modules, containing 195 genes, were positively associated with FOLFOX chemotherapy resistance (Figure 1B) and we selected 44 key genes with a MM value greater than 0.7. Next, we performed WGCNA analysis for GSE81653 using the same methods and parameters as previously to explore the relevant factors of recurrence risk after FOLFOX therapy which resulted in clustering of 7 co-expression modules (Figure 1C). The Mebrown and Meblue modules consisting of a total of 216 genes were positively associated with recurrence risk (Figure 1D) and we again selected 114 hub genes with a MM value higher than 0.7. We then intersected the key gene members from both WGCNA analyses corresponding to FOLFOX resistance and tumor recurrence which identified the complement component 3 (C3) gene as the only common element (Figure 1E).

### C3 Links the Subnetworks Contributing to Poor Prognosis of Colorectal Cancer

**FIGURE 2:**
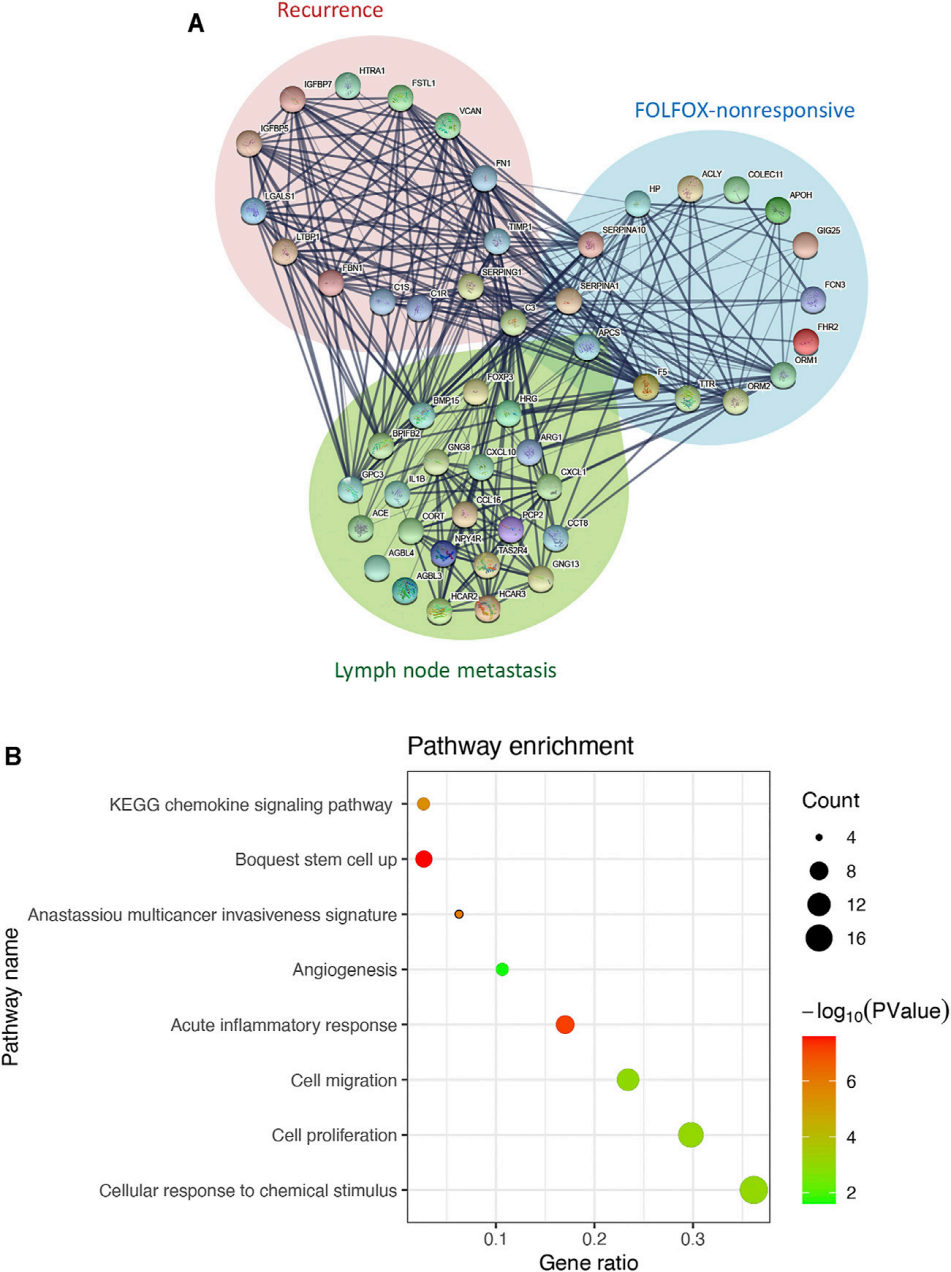
C3 interacts with genes associated with poor prognosis of colorectal cancer. **(A)** The interaction network of C3. Genes from significant modules related FOLFOX chemotherapy resistance are shown in the blue circle, genes from the significant module related to colorectal cancer recurrence are shown in the in red circle, and genes upregulated in colorectal cancer samples with lymph node metastasis are shown in the green circle **(B)** Enrichment analysis for genes in the C3 gene interaction network. A raw *p*-value less than 0.05 indicated statistical significance.

We then constructed a protein-protein interaction network by integrating the interaction relationships of the significant modules from the WGCNA analyses above and the differentially expressed genes between samples with and without lymph node metastasis to delineate the core genes connecting subnetworks responsible for FOLFOX chemotherapy resistance, tumor recurrence, and spread to lymph nodes. As highlighted in the interaction network (Figure 2A), the C3 gene represents one of the bridge hubs linking the three subnetworks. Further functional annotation analysis identified a significant enrichment of gene sets related to cancer stemness, invasiveness, migration, proliferation, angiogenesis, and inflammation response. In addition, cellular responses to chemical stimulus, a pathway associated with chemotherapy resistance, was also significantly enriched (Figure 2B). Based on these results, we propose that the C3 gene may act as a potential regulator of the prognostic CRC network and serves as a shared hub gene, rather than as a node with scattered functional edges participating in a single signaling pathway.

### High C3 Gene Expression Leads to Increased Colorectal Cancer Recurrence Risk and Poor Survival

**FIGURE 3:**
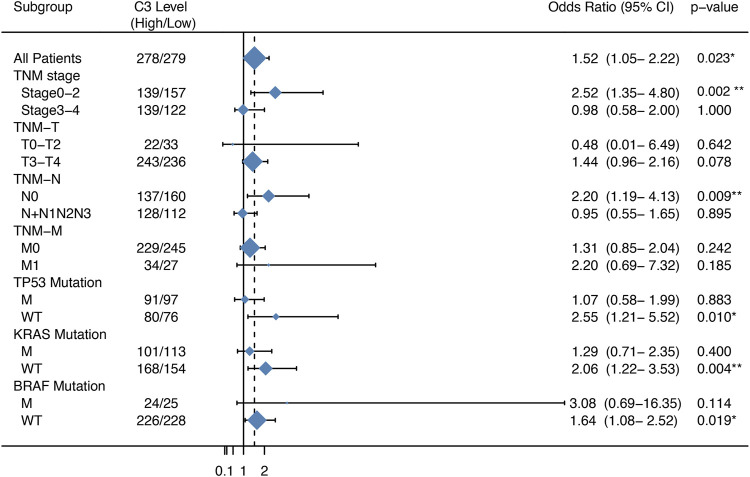
Forest plot of the association between C3 gene expression and the recurrence risk of colorectal cancer. Samples in the GSE39582 dataset were categorized according to the median expression C3 gene. Odds ratio (OR) and 95% confidence interval (95% CI) were calculated to assess the risk of recurrence. M: mutation; WT: wild type. *p < 0.05, **p < 0.01.

We next compared the effect of high and low expression of C3 on tumor recurrence in patients with CRC in the GSE39582 dataset. Patients with high C3 expression exhibited an increased risk of recurrence (OR = 1.52, 95% CI: 1.05–2.22; *p* = 0.023) (Figure 3). Further subgroup analysis showed that the levels of C3 expression had significantly positive effect on colorectal cancer recurrence in patients during early TNM stages (*p* = 0.002) and those without spread to lymph nodes (*p* = 0.009). In addition, we found a statistically significant susceptibility in subgroups without mutations in *TP53* (*p* = 0.010), *KRAS* (*p* = 0.004), and *BRAF* (*p* = 0.019).

**FIGURE 4:**
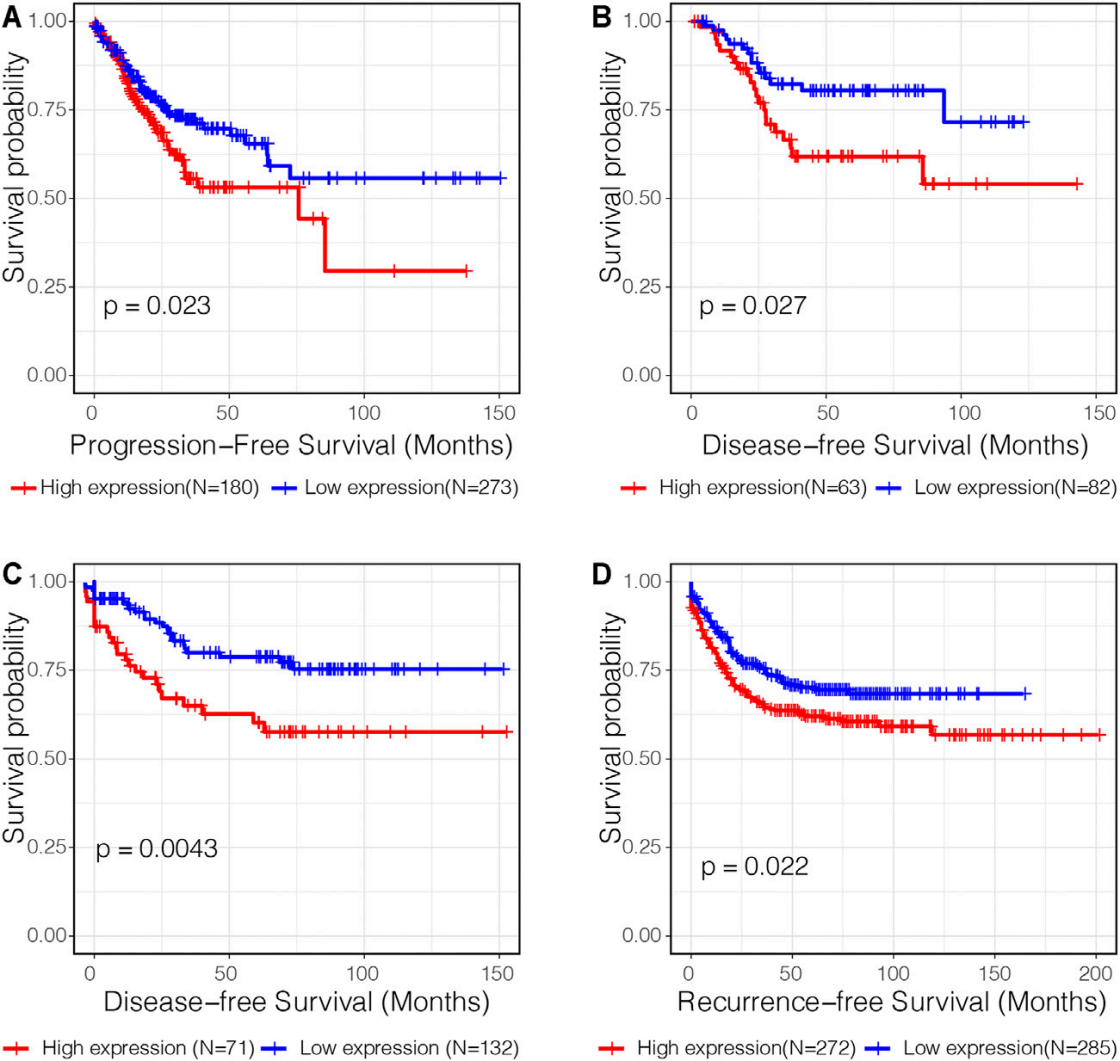
C3 gene expression is a significant prognostic factor of colorectal cancer. Kaplan-Meier curves assessing progression-free survival in the TCGA-COAD dataset **(A)**, disease-free survival in the GSE17536 dataset **(B)** and GSE87211 dataset **(C)**, and recurrence-free survival in the GSE39582 dataset **(D)** were plotted. Optimal separation based on C3 gene expression for samples in each dataset was identified to achieve best statistical significance. A *p*-value less than 0.05 indicated statistical significance.

Subsequent survival analysis was performed to validate the prognostic value of C3 in four independent transcriptome datasets with a total of 1,358 samples. Kaplan-Meier plots revealed that high C3 expression was associated with a statistically significant unfavorable progression−free survival (Figure 4A, *p* = 0.023), disease−free survival (Figures 4B,C; both *p*-values < 0.05), and recurrence−free survival (Figure 4D, *p* = 0.022). Therefore, we propose that C3 represents a candidate biomarker for poor prognosis in colorectal cancer patients.

### C3 Overexpression Promotes Oxaliplatin Resistance

**FIGURE 5:**
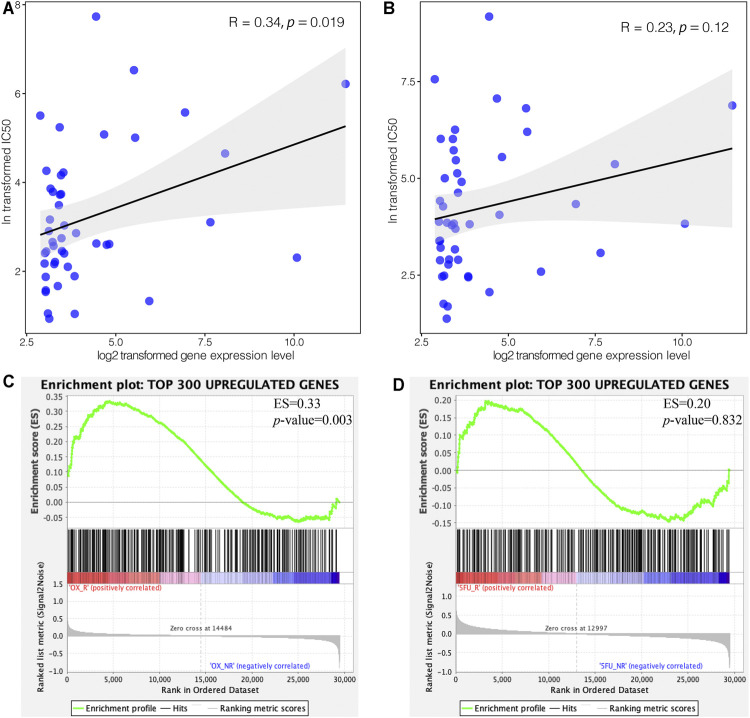
High expression of C3 induces resistance to oxaliplatin but not 5-Fluorouracil. Pearson correlation test revealed a statistically significant association of C3 gene expression with the corresponding IC50 of oxaliplatin **(A)**, but not 5-Fluorouracil **(B),** in colorectal cancer cell lines. GSEA demonstrated that the top 300 upregulated genes in colorectal cancers with high C3 expression were significantly enriched in Oxaliplatin resistant cells **(C)**, but not in 5-Fluorouracil resistant cells **(D)**. A raw *p*-value less than 0.05 indicated statistical significance.

To explore the impact of C3 gene expression on resistance to FOLFOX chemotherapy, we assessed the correlation between C3 gene expression in different colorectal cancer cell lines and the corresponding IC50 of 5-Fluorouracil and Oxaliplatin. C3 gene expression was positively associated with Oxaliplatin IC50 (Figure 5 **A**, *p* = 0.019), but not with 5-Fluorouracil (Figure 5B, *p* = 0.120). Next, we compared the transcriptome concordance between chemotherapy reagent resistance and C3 overexpression. The top 300 up-regulated genes were obtained by comparing the samples with a high and low abundance of C3 expression (no significantly down-regulated gene were identified because of insufficient log2-transformed fold changes). The GSEA results found these genes were significantly enriched in the comparison between Oxaliplatin-resistant cells and naïve cells (Figure 5C, *p* = 0.003). However, the enrichment was not statistically significant in the cell model of 5-Fluorouracil resistance (Figure 5D, *p* = 0.832). This was consistent with the results of *in silico* IC50 tests. Based on the observations above, it could be reasonably speculated that a high C3 gene expression in colorectal cancer might promote resistance to Oxaliplatin.

### High C3 Gene Expression Shapes the Immune Microenvironment of Colorectal Cancer

**FIGURE 6:**
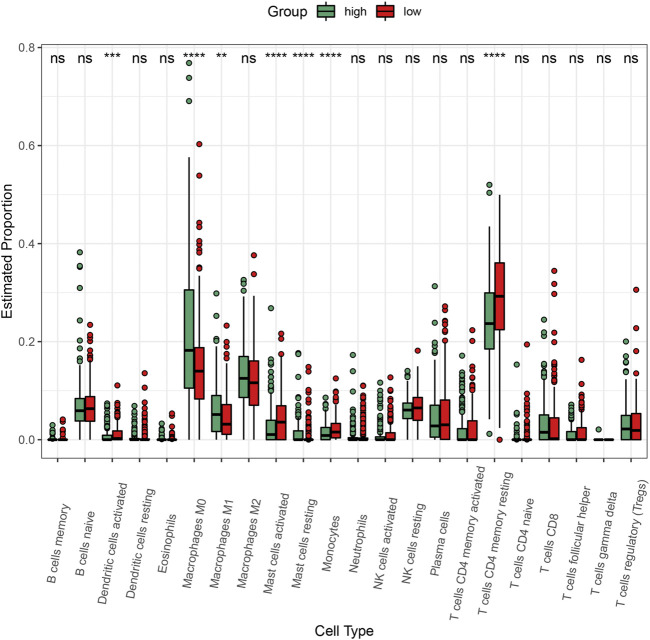
Comparison of the abundance of infiltrating immune cells between high-C3 and low-C3 groups in the TCGA-COAD dataset. Samples were categorized according to the median expression level of C3 gene. Kruskal-Wallis test was performed to statistically compare differences. *p < 0.05, **p < 0.01, ***p < 0.001, ****p < 0.0001.

To explore the impact of C3 gene expression on the tumor immune microenvironment, we employed the CIBERSORT algorithm to compare the abundance of 22 human infiltrating immune cell types between colorectal cancers with C3 gene expression above and below median level (Figure 6). This revealed a large heterogeneity of infiltrating immune cells between the two different C3 gene expression groups. There were significantly more infiltrating dendritic cells activated, mast cells activated, monocytes, and T cells CD4 memory resting in colorectal cancers with C3 lower expression compared to those with higher C3 expression (all *p*-values < 0.001). Conversely, tumors with high C3 expression showed significantly higher fractions of M0 macrophages, M1 macrophages, and mast cells resting (all *p*-values < 0.01). No significant difference of other immune cells fraction was found (all *p*-values > 0.05).

### C3 Overexpression Drives Key Signaling Cascades Regulating Colorectal Cancer Progression

**FIGURE 7:**
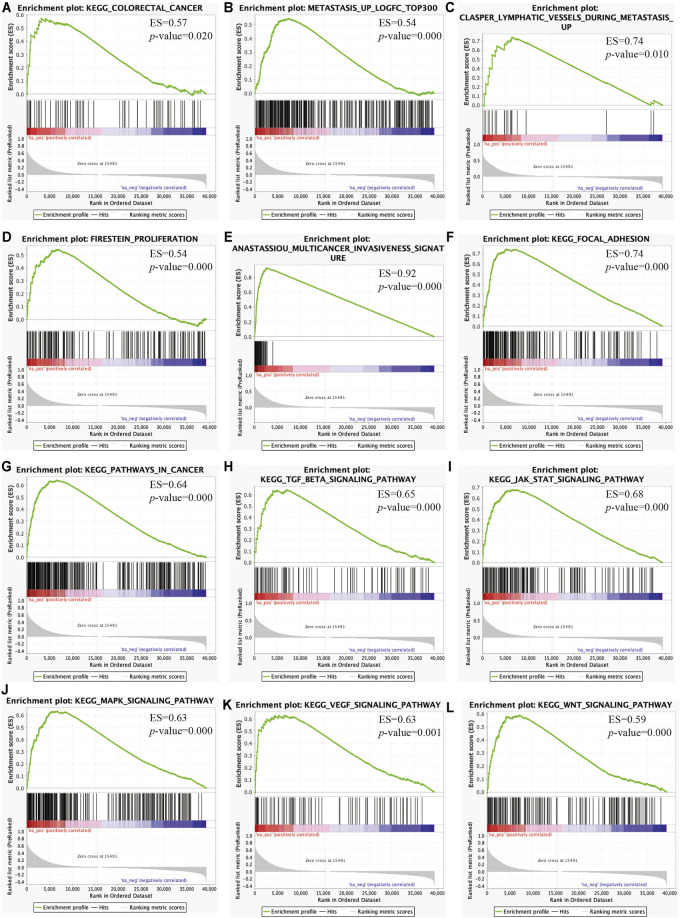
Identification of key pathways and gene sets associated with colorectal cancer prognosis based on TCGA-COAD RNA-Seq dataset via gene set enrichment analysis (GSEA). Gene sets for colorectal cancer **(A)**, lymphatic metastasis **(B)**, lymphatic vessels during metastasis **(C)**, proliferation **(D)**, multicancer invasiveness signature **(E)**, focal adhesion **(F)**, pathways in cancer **(G)**, TGF-BETA signaling **(H),** JAK-STAT signaling **(I)**, MAPK signaling **(J),** VEGF signaling **(K)**, and WNT signaling **(L)** were significantly enriched. Samples were categorized according to the median expression level of C3 gene. A raw *p*-value less than 0.05 indicated statistical significance.

We then performed GSEA to elucidate the biological pathways regulated by C3 that are involved in the progression of CRC. This revealed that clinical CRC samples with higher C3 expression were enriched for the general pathway of colorectal cancer development and progression (Figure 7A). Furthermore, lymphatic metastasis signatures identified by our microarray data were also significantly overrepresented (Figure 7B) and likewise, genes associated with invasion of lymphatic vessels during metastasis also showed marked enrichment (Figure 7C). In addition, further gene set mining suggested that higher C3 expression could promote cancer proliferation (Figure 7D), invasion (Figure 7E), and adhesion (Figure 7F). Taken together, these observations corroborated the hypothesis that a higher C3 gene expression could drive poor prognosis of colorectal cancer. To further uncover the underlying mechanisms, we next investigated alterations of biological pathways upon C3 hyperexpression. Colorectal cancers with high C3 gene level exhibited a hyperactivation of several pathways including pathways in cancer (Figure 7G), TGF-BETA signaling (Figure 7H), JAK-STAT signaling (Figure 7I), MAPK signaling (Figure 7J), VEGF signaling (Figure 7K), and WNT signaling (Figure 7L).

To summarize, overexpression C3 gene is likely to activate several signaling pathways and plays a prominent role in initiating cancerous deterioration, driving poor prognosis.

## Discussion

Surgery and radiation are curative options for patients during early stages of CRC. Conversely, CRC patients at advanced stages typically receive chemotherapy to reduce the rate of recurrence and improve the survival rate. Oxaliplatin, a third-generation platinum-based anticancer agents, is approved in combination with fluorouracil (5-FU) or leucovorin (LV) for advanced CRC treatment (Mandala et al., 2004; Yothers, 2012). Although certain CRC patients typically benefit from treatment with oxaliplatin, many acquire resistance and thus their prognosis can be poor (Culy et al., 2000). It is therefore of great importance to identify potential biomarkers for oxaliplatin resistance in order to facilitate treatment decisions. In this study, we identified complement component 3 (C3) as a critical gene associated with FOLFOX chemotherapy resistance by integration and analysis of public transcriptomic data and our own microarray data.

C3 is a key molecule of the enzymatic complement cascade reaction which acts as a powerful pro-inflammatory factor. It can activate a variety of signaling pathways, such as NF-κB, IRF, AP-1 pathway, resulting in inflammation (Tam et al., 2014). Studies have shown that C3 acts as a potential biomarker for inflammation and immunity-related disease. For examples, levels of plasma C3 are significantly elevated in patients with rheumatoid arthritis and axial spondyloarthritis (Arias de la Rosa et al., 2020). Likewise, C3 levels were significantly upregulated in the mucosae of patients with inflammatory bowel disease (Sugihara et al., 2010). Inflammation and tumor often go hand in hand, and chronic inflammation can alter the microenvironment in favor of tumor formation and growth (Balkwill and Mantovani, 2001)*.* Moreover, the complement can increase the production of tumor growth factors, inhibit apoptosis, promote angiogenesis, and inhibit anti-tumor immunity to promote oncogenesis (Rutkowski et al., 2010). C3 has previously been shown to play critical roles in several cancer types, including glioblastoma, ovarian cancer, skin squamous cell carcinoma, and CRC. For instance, deposition of C3 has been found to be higher in glioblastoma than in controls (Bouwens et al., 2015). Ovarian cancer cells can secret C3, which promotes tumor growth and metastasis (Cho et al., 2014; Cho et al., 2016). C3 is upregulated in skin squamous cell carcinoma and promotes the growth of cutaneous squamous cell carcinoma (Riihila et al., 2017). Plasma C3 levels have previously been shown to be significantly increased in CRC (Dowling et al., 2012). In a mouse model of colorectal tumor transplantation, complement inhibition and complement depletion were used to reduce the plasma levels C3 and it was found that a reduction of C3 levels inhibited tumor growth, while tumor progression was resumed upon replenishment of C3 (Downs-Canner et al., 2016). The abovementioned studies have provided important evidence about the oncogenic function of C3. However, the potential role and clinical application of C3 in CRC had remained largely undefined. Here, we demonstrate that C3 is a key gene associated with FOLFOX chemotherapy resistance and cancer recurrence risk. The prognostic power of C3 for CRC is supported by the clinical data showing that high C3 expression correlates with an unfavourable prognosis as evidenced by poor progression-free survival and disease-free survival as well as an increased CRC recurrence risk. Thus, C3 expression levels could be used as an index for predicting the outcome of CRC chemotherapy in future clinical studies.

Mechanisms of oxaliplatin resistance are complex and involve many molecular events including reduced drug uptake, decreased apoptotic response, and enhanced tumor immunosuppressive effect (Martinez-Balibrea et al., 2015; Ning et al., 2021). There is conflicting evidence regarding the mechanism of oxaliplatin resistance in cancer. On one hand, oxaliplatin treatment results in an increased proportion of CD4^+^/CD8^+^ T lymphocytes and decreased proportion of regulatory T cells (Tregs) (Stojanovska et al., 2019). Conversely, an increased proportion of Tregs has also previously been observed in peripheral blood of CRC patients that received combined oxaliplatin and 5-FU treatment (Vizio et al., 2012). In this study, we found a large heterogeneity of infiltrating immune cells. Patients with different C3 expression exhibited significantly differential infiltration of activated dendritic cells and mast cells, mast cells resting, M0 and M1 macrophages, monocytes, and resting CD4^+^ memory T cell. GSEA results further showed that gene signatures representing oncogenic signaling and metastasis, such as pathways involved in cancer, TGFβ signaling, JAK-STAT signaling, MAPK signaling, VEGF signaling, and WNT signaling, were all significantly enriched in the patients with higher C3 expression compared to those with lower C3 expression. Taken together, these data indicate that overexpression of C3 confers CRC cells resistance to oxaliplatin treatment, The resistance mechanism may likely involve a reprogramming of the tumor immune microenvironment and oncogenic signaling.

In summary, we identify C3 as an oxaliplatin resistance-related gene using both public datasets and our own microarray data. These findings highlight a potential clinical role of C3 as a predictive biomarker for oxaliplatin-based therapy response and provide a theoretical guidance and decision-making for oxaliplatin-based chemotherapy in CRC patients.

## Data Availability

The datasets presented in this study can be found in online repositories. The names of the repository/repositories and accession number(s) can be found below: https://ngdc.cncb.ac.cn/omix/release/OMIX505
